# Molecular Genetic Analysis of Human Endometrial Mesenchymal Stem Cells That Survived Sublethal Heat Shock

**DOI:** 10.1155/2017/2362630

**Published:** 2017-12-10

**Authors:** A. E. Vinogradov, M. A. Shilina, O. V. Anatskaya, L. L. Alekseenko, I. I. Fridlyanskaya, A. Krasnenko, A. Kim, D. Korostin, V. Ilynsky, A. Elmuratov, O. Tsyganov, T. M. Grinchuk, N. N. Nikolsky

**Affiliations:** ^1^Institute of Cytology, Russian Academy of Sciences, Tikhoretskay Ave 4, St. Petersburg 194064, Russia; ^2^Medical Genetics Centre Genotek, Nastavnichesky Alley 17-1-15, Moscow 10510, Russia; ^3^Vavilov Institute of General Genetics, Russian Academy of Sciences, Gubkina Str. 3, Moscow 119333, Russia

## Abstract

High temperature is a critical environmental and personal factor. Although heat shock is a well-studied biological phenomenon, hyperthermia response of stem cells is poorly understood. Previously, we demonstrated that sublethal heat shock induced premature senescence in human endometrial mesenchymal stem cells (eMSC). This study aimed to investigate the fate of eMSC-survived sublethal heat shock (SHS) with special emphasis on their genetic stability and possible malignant transformation using methods of classic and molecular karyotyping, next-generation sequencing, and transcriptome functional analysis. G-banding revealed random chromosome breakages and aneuploidy in the SHS-treated eMSC. Molecular karyotyping found no genomic imbalance in these cells. Gene module and protein interaction network analysis of mRNA sequencing data showed that compared to untreated cells, SHS-survived progeny revealed some difference in gene expression. However, no hallmarks of cancer were found. Our data identified downregulation of oncogenic signaling, upregulation of tumor-suppressing and prosenescence signaling, induction of mismatch, and excision DNA repair. The common feature of heated eMSC is the silence of MYC, AKT1/PKB oncogenes, and hTERT telomerase. Overall, our data indicate that despite genetic instability, SHS-survived eMSC do not undergo transformation. After long-term cultivation, these cells like their unheated counterparts enter replicative senescence and die.

## 1. Introduction

Mesenchymal stem cells (MSC) are self-renewing multipotent cells, which hold a great potential in regenerative medicine and tissue engineering reflected by more than 500 MSC-based clinical trials registered with the NIH [1, 2]. MSC were isolated from multiple sources, such as bone marrow, adipose tissue, blood vessel walls, peripheral and umbilical cord blood, Wharton's jelly, and Fallopian tubes.

Currently, the MSC of endometrium (eMSC) attract growing attention. Comparing with other MSC types, eMSC show a higher vasculogenic and anti-inflammatory potential [3]. These valuable features are associated with a special role of eMSC in every month endometrium growth [3]. Cultured eMSC are being applied in clinical trials, and encouraging results have been reported [4].

Typically, to accumulate clinically relevant cell mass, isolated stem cells should be expanded in vitro. An important point to consider is the genetic stability of stem cells during long-term cultivation. Genome stability ensures oncogenic safety and is a crucial risk factor in stem cell-based therapies. However, the literature data concerning the genome maintenance during prolonged cultivation of human MSC are ambiguous. Mounting evidence indicates that long-term MSC expansion may be accompanied with an occurrence of chromosomal abnormalities [5, 6]. It is well known that chromosome abnormalities boost the tumor development. However, MSC even with chromosomal alterations showed progressive growth arrest and entered replicative senescence during prolonged cultivation. Currently, there are no studies reporting transformation of human MSC during long-term culturing in vitro even despite genome instability [5, 6]. Some papers on spontaneous malignant transformation [7, 8] were later retracted from publication. Using DNA fingerprinting and/or short tandem repeat analysis to compare “transformed” and normal MSC, it was found that in reality, “transformed” cells were crosscontaminated with cells of various permanent cell lines [9]. Nonetheless, some authors suggest that spontaneous malignant transformation of human MSC is not completely excluded [10]. Long-term cultivation of bone marrow- and liver-derived MSC produced transformed cells with tumorigenic potential. High-resolution genome-wide DNA array and short tandem repeat profiling confirmed a shared origin of the transformed cells and parental MSC [10]. The results of this publication have not yet been confirmed.

Stress response of stem cells is under active investigation [11]. However, the fate of stem cells cultivated after exposure to damaging factors is poorly monitored. Hyperthermia is an important ecological and therapeutic factor. Heat shock (HS) response has been studied for decades. The research was mainly focused on the expression of heat shock proteins (HSP) and heat shock factors (HSF) as well as their involvement in the regulation of various cellular functions. Traditionally, it was considered as a nonmutagen, that is, the agent not inducing DNA damage. Recently, it becomes clear that HS induces DNA damage and affects DNA integrity [12]. It was reported that HS-induced chromosomal instability in cancer cells [13]. HS response of human MSC is badly studied. Usually, HS is considered as an inductor of apoptosis or necrosis. The results of our studies demonstrated that eMSC unlike embryonic stem cells responded to sublethal temperature by stress-induced premature senescence (SIPS) [14, 15]. Treated cells exhibited gamma-H2AX-foci. It points to DNA damage as the appearance of gamma-H2AX-foci is a marker for DNA double-strand breaks.

The ability of HS to generate DNA damage makes it a convenient tool for the investigation of safety margins related to eMSC genome instability and transformation. To be appropriate for preclinical and clinical trials, a biological product (eMSC in our case) should have wide safety margins [16]. Since MSC transplantation is often accompanied by inflammatory processes triggering fever and hyperthermia in patients, we aimed to investigate genomic stability and possibility of malignant transformation in eMSC that stayed alive after SHS. SHS was induced at 45°C during 30 minutes. This particular design of treatment was chosen because in contrast to cells in vivo that can live maximum at 41-42°C [17], cultured cells may have maximal temperature threshold at 45.5°C [14]. It should be noticed that the minimum temperature producing a burn is 44°C but injured tissues are able to recover. In this study, we used methods of classic and molecular karyotyping, next-generation sequencing, and transcriptome functional analysis. Also, we compared our results with published data on gene expression changes in hTERT-transformed human bone marrow mesenchymal stem cells [18].

## 2. Materials and Methods

### 2.1. Cells and Treatment

The study was performed on eMSC derived from desquamated endometrium in the menstrual blood [19]. The cells were maintained in DMEM/F12 medium (Gibco, United States) with 10% bovine fetal serum (HyClone, United States), 1% antibiotic-antimycotic solution, and 1% GlutaMAXTM (Gibco, United States). The cells were subcultured 1 : 3 twice a week using 0.05% trypsin with EDTA (Invitrogen, United States).

### 2.2. HS Conditions

eMSC exposed to SHS were plated in 3 cm Petri dishes at density 10^5^ cells/plate. The next day, dishes sealed with Parafilm were submerged into water bath at 45°C for 30 min and then returned to 37°C in CO_2_ incubator. Survived cells (about 3 × 10^4^ cells) were plated in a 3 cm Petri dish after 3 days. Upon reaching the monolayer, these cells were subcultured under the same conditions as intact cells. At the 6th passage after HS, the cells were analyzed using classic and molecular karyotyping, next-generation sequencing, and transcriptome and functional analysis.

### 2.3. G-Banded Karyotyping

The cells were seeded with a density of 14-15 × 10^3^ cells/cm^2^. Mitostatic agent colcemid (Sigma, United States) in dose 30 *μ*g/mL was added after 24-25 h for 1 h. Then the medium was removed and the cells were harvested with 0.05% trypsin and centrifuged. The pellet was suspended and treated with 0.56% KCl hypotonic solution for about 1 h. The cell suspension was centrifuged (1300 rpm), resuspended, and fixed on ice by methanol mixed with acetic acid in the ratio 3 : 1. The fixative was changed three times, the total fixation time being 1.5 h. The fixed material was dropped onto cold and wet slides. The slides were air-dried for 1 week. Chromosomes were G-banded with Giemsa stain (Fluka, United States) after preliminary trypsinization. Metaphase plates with well-spread chromosomes were assayed under an Axio Scope microscope (Carl Zeiss, Germany). The chromosomes were identified according to the international nomenclature [20]. A total of 65 metaphases (31 of intact and 34 of SHS-survived eMSC) were analyzed.

### 2.4. Molecular Karyotyping

It was carried out in Genoanalitica (Moscow, Russia) using HumanCytoSNP-12 kit (Illumina®, United States) according to the manufacturer's recommendations. They were seeded with a density of 3 × 105 cells per 6 cm plate and lysed in 72 h for DNA isolation. DNA preparation, hybridization, washing, staining, and sample scanning were done according to the standard protocols of the company Illumina. The samples were hybridized on high-density oligonucleotide arrays with 3 × 105 isothermic probe-covered unique gene and intergenic areas of the human genome. The scanning was done with iScan (Illumina, United States). The results were analyzed with Genome Studio Genotyping Module and BlueFuse software (Illumina, United States).

### 2.5. SA-*β*-Gal Assay

Cell staining for *β*-galactosidase (*β*-gal) activity was performed using Senescence *β*-Galactosidase Staining Kit (Cell Signaling Technology) according to the manufacturer's protocol and quantified microscopically by counting X-gal-positive cells among not less 500 cells.

### 2.6. Next-Generation Sequencing

Sample preparation for NGS and sequencing on the Illumina platform were performed in Genotek company (Moscow, Russia). RNA was extracted using Pure Link RNA Mini Kit (AMBION, Life Technologies). After that, mRNA was extracted from total RNA using magnetic beads (Sileks). cDNA libraries were prepared using NEBNext® mRNA Library Prep Reagent Set for Illumina (New England Biolabs). In this approach, mRNA was fragmented, cDNA was synthesized, end repaired, and ligated to unique sequencing adaptors to form cDNA libraries. Dual indexing was performed by PCR with NEBNext Multiplex Oligos for Illumina (dual index primers set 1). Quality control of prepared libraries was made using Bioanalyzer 2100 (Agilent Technologies). Sequencing of cDNA libraries was done on an HiSeq2500 (Illumina) in rapid run mode with read length 100 nt.

Next-generation sequencing was done by parallel measurement of three biological samples both for control and SHS-treated eMSC. The NGS reads were trimmed using trimmomatic software specially developed for Illumina NGS data, with default parameters [21]. The trimmed reads were mapped to canonical nonredundant human transcriptome presented in RefSeq database [22] using Bowtie 2 software [23, 24]. This aligner became a de facto standard within mapping pipelines and shows a remarkable tolerance both to sequencing errors and indels [25]. Bowtie 2 was used with the “very sensitive” preset of parameters, which allows the most sensitive and accurate mapping (at the expense of speed). Only the nonambiguous mappings were counted.

The differential expression was determined as in the previous work [26]. The obtained counts were analyzed using the “limma” package (implemented in *R* environment) specially developed for whole-transcriptome analyses of differentially expressed genes [27]. Comparison of different software packages showed that limma is the method of choice for our goals [28]. Taking into account recommendation in limma manual, genes with counts below 10 in all probes were discarded. This procedure gave 10,880 genes for analysis of differential gene expression. The data normalization methods presented in limma (quantile, scale) were tested as well as the trimmed mean method from the edgeR package [29]. The results were similar. The results obtained with quantile normalization are shown.

### 2.7. Gene Module Analysis

The biological processes and molecular pathways enriched in differentially expressed genes were found similar to previous works [30, 31]. The biological processes were taken from GO database [32]. For each GO category (process), all its subcategories were collected using GO acyclic directed graphs, and a gene was regarded as belonging to a given category if it was mapped to any of its subcategories. As a source of molecular pathways, the NCBI BioSystem was used, which is the most complete compendium of molecular pathways from different databases [33]. The redundancy was removed by uniting entries with identical gene sets.

The hypergeometric distribution of probability (implemented in *R* environment) was used for determination of statistical significance of observed-to-expected ratios of gene numbers in different biological processes or molecular pathways in the gene samples (as compared to total gene set). The contrast test was used for analysis of gene expression folds. In this test, the mean expression fold of genes belonging to each process/pathway is compared with the mean fold of the total gene set. For evaluation of two-tailed statistical significance of an obtained contrast between these folds, 20,000 random samplings were taken from total gene set (of a size equal to the number of genes in a process/pathway). This method is preferable to parametric or nonparametric tests because normal distribution that is required for parametric tests is usually absent, whereas nonparametric tests can lose a considerable amount of information. The random-sampling test is distribution independent and retains all information. In both hypergeometric test and contrast test, the adjustment of obtained *p* values for multiple comparisons was done according to the method by Storey and Tibshirani [34]. This procedure gives *q* value, which can be considered as *p* value corrected for multiple tests.

### 2.8. Protein-Protein Interaction Network Construction and Analysis

The protein-protein interactions (PPI) were taken from the STRING database [35]. We choose this database because STRING places its focus on functional relationship between two proteins, contributing to a common biological purpose and contains interactions from multiple sources: experimental interactions imported from primary databases, pathways from manually curated databases, and statistical and semantic links between proteins, obtained from Medline abstracts and a large collection of articles [35].

The PPI networks were visualized also using the STRING server. We analyzed dense-connected components of protein-protein interaction networks for proteins encoded by genes demonstrating expression difference between SHS-survived and control eMSC by more than 8-folds (the top one-quarter of genes with the most differing expression). The induced and inhibited proteins were analyzed separately.

### 2.9. The Identification of Hub Proteins That Are Causal Regulators in Modular and Network Organization

Protein interaction network consists of nodes and edges, where each node stands for a protein and the edges represent interactions [36]. The number of edges per node characterizing the number of interacting proteins is termed a degree. Nodes with the highest degree are defined as hubs [37]. The degree is a fundamental parameter that is usually adopted to evaluate the nodes in a network for the identification of evolutionary conserved causal regulators in modular organization and networks [38, 39]. As recommended by Han et al. [40], nodes with degree greater than 5 were labeled as hubs.

### 2.10. PPI Network and Cluster Analysis

Biological networks are composed by subnetworks implicated in various biological processes. To identify these subnetworks, we applied K-means clustering using the STRING sever. After the clustering, function annotation of clusters was made. The function annotation included GO (gene ontology) analysis and KEGG (Kyoto encyclopedia of genes and genomes) pathway analysis. The Benjamini method was used to control the false discovery rate (FDR) to correct the *p* value.

## 3. Results and Discussion

### 3.1. SHS Triggers Chromosome Instability in eMSC

In this study, we applied HS induction at 45°C during 30 minutes. This temperature is used in clinical practice for local and regional hyperthermia [41]. Clinical hyperthermia involves temperature elevation in the range of 39–45°C. Temperatures above 45°C sustained for more than several minutes usually lead to protein denaturation and cell death typically via necrosis. It is known that mesenchymal stem cells are present in many tissues of an adult organism and thus heating can lead to cell damage. This design of heating was used in a large number of studies since this treatment is sublethal and effectively induces a cellular response to heat stress [12, 14, 15]. Our previous studies performed with the same SHS induction design demonstrated that SHS triggered premature senescence of eMSC in culture. It was found that SHS-induced DNA damage response displayed in appearance of gamma-H2AX-foci. Nevertheless, 35% of cells were alive after SHS. Survived cells maintained key stem cell properties, including self-renewal features, CD marker expression pattern, and differentiation capacity [15]. Thus, SHS model “45°C during 30 minutes” is a very convenient one for the evaluation of tumorigenic potential with wide safety margins.

**Figure 1:**
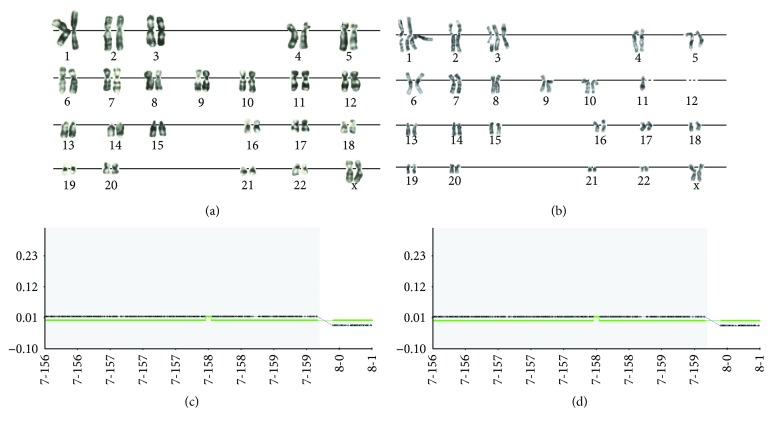
Karyotype analysis of eMSC. (a) G-banded karyotype of intact eMSC. (b) G-banded karyotype of SHS-survived eMSC. The picture illustrates trisomy and near-centromere breakage of chromosomes 1, 3, monosomy of chromosomes 11; absence of 2 homologs of chromosome 12 is seen in cells after SHS. (c) Molecular karyotyping of intact eMSC; (d) Molecular karyotyping of SHS-survived eMSC; *abscissa*: length of chromosome 7 in genome, rel. units; *ordinate*: logarithm of signal intensity, rel. units; *arrow* shows duplication in chromosomes 7, locus 7q36.3, and size 62 kB; *dots on black solid line*: deviations in each single base-pair difference in the DNA sequence according to the signal intensity; *green solid line*: deviations in single base-pair difference in the DNA sequence from the norm with signal intensity combined with genotype.

In this study, we assessed chromosome stability in eMSC that survived SHS using G-banding and molecular karyotyping. G-banding of these cells revealed random structural and functional chromosome abnormalities (aneuploidy, chromosome breakages) (Figure 1(b)). Control eMSC maintained the normal karyotype (Figure 1(a)). In contrast to G-banding, the results of molecular karyotyping performed with microarrays did not reveal the difference between intact and SHS-treated eMSC cells. The analysis identified in both intact and SHS-survived cell small duplicated fragment (62 kB) in the chromosome 7 (7q36.3) signifying that the defect is of the donor origin (Figures 1(c) and 1(d)). The comparison of the data obtained by G-banding and molecular karyotyping suggests that although hyperthermia disturbs karyotype structure, stress-related chromosome rearrangements are random. Karyotype destabilization was observed in immortalized Chinese hamster fibroblasts after long-term cultivation under mild hyperthermia [42] as well as in mouse embryonal carcinoma exposed to severe heat stress [13].

The inconsistency between G-banding and molecular karyotyping may originate from their different focuses. G-banding visualizes only large-scale genetic instability, including aneuploidy, chromosome breakages, and chromosome rearrangements (deletions and insertions) that involve more than 5 Mb [43]. The advantage of this method is that it works with high accuracy even at the level of one cell [44]. Also, G-banding still is the only tool for the detection of balanced rearrangements (i.e., that do not change gene dosage balance) and low-level mosaicism, both of which are not detectable by molecular karyotyping [45]. Molecular karyotyping or “chromosomal microarray” evaluates both large- and small-scale rearrangements covering 0.5–1.0 Mb, including single-nucleotide polymorphism (SNP) and gene copy number variations. However, clinical sensitivity of molecular karyotyping depends on the proportion of potentially pathogenic rearrangements because it reveals pathogenic changes only if they occur in no less than 10% of all analyzed cells [44].

In our study, the abnormalities detected by molecular karyotyping were not detected by G-banding because of their small size. For example, the loss of heterozygosity, identified by molecular karyotyping, was not detected by G-banding. In turn, the failure of the identification of chromosome breakages by molecular karyotyping may originate from the occurrence of breakages at different chromosomal loci and from the preservation of the amount of genetic material. Also, molecular karyotyping did not confirm trisomy and monosomy because both abnormalities were observed in the number of cells less than the threshold sensitivity of the molecular karyotyping method. Thus, a comparison of the data of G-banding and molecular karyotyping provided a detailed description of eMSC karyotype at various levels of genome organization.

Molecular mechanisms of hyperthermia-related chromosome instability have been little investigated. Currently, we know that SHS response is rapid and dynamic and results in induction of several hundred and repression of several thousand genes [46]. The master regulators of SHS response are transcription regulator HSF and chaperones of HSP family. It was recently shown that stressful conditions impair the ability of these regulators to appropriately coordinate pathways of DNA repair, cytoskeleton maintenance, and chromosome segregation [47–49]. As a result, HSF1 and HSP90 function impairment may promote genetic instability [49].

Karyotypic changes are closely linked with the cancer development and tumor progression [50]. Recent research highlighted that cancer risk is heavily influenced by extrinsic factors [51]. Chromosomal rearrangements observed in SHS-treated cells pose the risk of their immortalization and malignancy. To investigate tumorigenic potential of these cells, we performed the entire transcriptome sequencing and bioinformatic analysis with particular attention to transformation-related traits.

### 3.2. SHS-Survived eMSC Transcriptomic Landscape

To obtain a genome-wide picture of gene activity difference in SHS-survived versus control eMSC, we performed NGS transcriptome sequencing and bioinformatic analysis of enriched molecular pathways for differentially expressed genes. A total of 8724 protein-coding genes were identified as differentially expressed at 2-fold threshold. Approximately, similar amounts of genes were induced or inhibited. 758 genes were upregulated and 729 genes were downregulated by more than 10-folds, whereas 4514 genes were upregulated and 4210 were downregulated by more than 2-folds. We did not reveal differential expression of the modules related to HS response coordinated by HSP proteins and HSF transcription factors.

**Figure 2:**
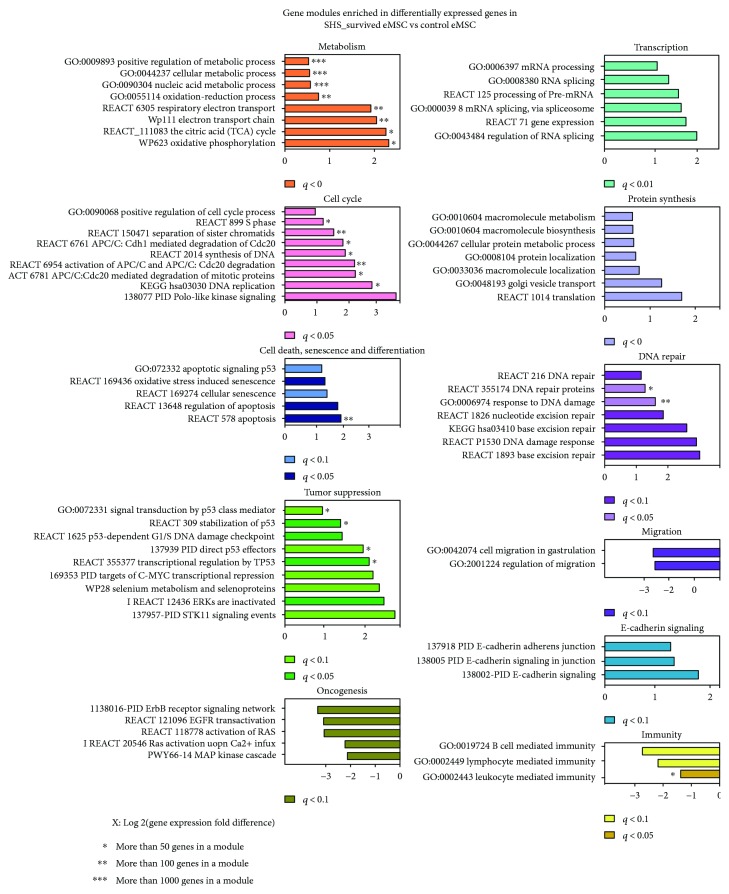
Gene modules that are induced or repressed in SHS-survived versus control eMSC with high significance (*q* < 0.1). This figure illustrates general metabolic activation in SHS-survived versus untreated eMSC seen from the upregulation of modules related to energy metabolism, cell cycle, protein turnover, DNA repair, and transcription. The figure shows increased tumor suppression that is evident from the upregulation of TP53 signaling, the induction of the module “targets of MYC transcriptional repression” uncovering the mechanism of Myc suppression in eMSC cells and from the decreased activity of oncogenic pathways.

Although fold change analysis of differentially expressed genes is the most commonly used approach for the identification of specific biological traits and potential biomarkers, this approach can overlook biologically meaningful molecules without large fold change such as transcription factors operating commonly at 20–150% expression difference [52]. To overcome this difficulty, we perform gene module analysis. For this propose, we selected GO categories and BioSystem pathways enriched for differentially expressed genes with high significance (*q* < 0.10). The selection yielded more than 100 gene modules (Figure 2). Surprisingly, about 85% of these modules were induced and only 15% were inhibited. The inconsistency between the numbers of activated and inhibited genes and the numbers of activated and inhibited gene modules (about 50% versus 50% and 85% versus 15%) suggests that the whole gene modules were activated in SHS-survived cells, whereas the inhibited genes were scattered among many modules achieving a level of significant enrichment only in a few of them.

The examination of functional distribution indicated that the induced modules related mostly to energy metabolism, transcription, mRNA splicing and processing, protein turnover, cell cycle, DNA repair, E-cadherin signaling, senescence, and tumor. The activation of modules related to energy metabolism, transcription, and protein turnover suggests that SHS-survived eMSC exhibited activated gene expression. The induction of modules implicated in the regulation of the first half of cell cycle may originate from unscheduled DNA synthesis that usually accompanies genome instability and manifestations of senescence [53]. The inhibited gene modules related lymphocyte mediated immunity involved in inflammation and cell migration (Figure 2) suggesting that SHS eMSC demonstrate better anti-inflammatory and stress-protecting properties compared to untreated cells.

The predominant induction of gene modules motivated us to investigate complex interplay between separate genes and functional modules using protein-protein interaction (PPI) network analysis. It is well established that compared to differential expressions of separate proteins and gene module analysis, PPI networks demonstrate better match between datasets and have an ability to provide more comprehensive insight to the data [54]. Also, in contrast to canonical pathways covering only a fraction of the true protein-protein interactions that occur within a cell, the networks can be constructed from extensive experimental data, the literature, and public databases of molecular interactions [55, 56].

**Figure 3:**
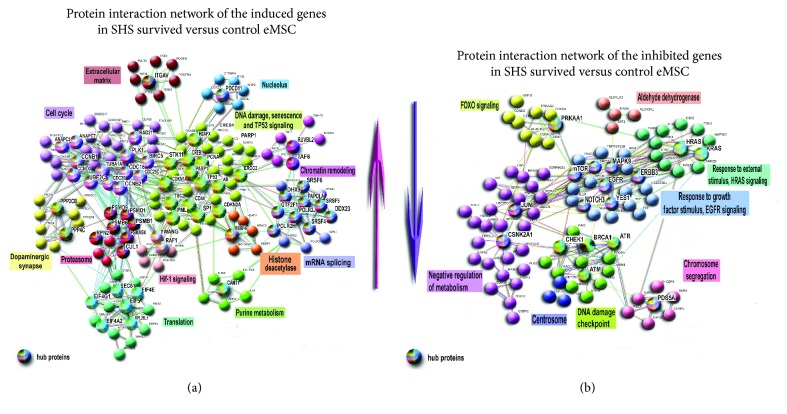
PPI networks for the most induced and inhibited genes in SHS-survived versus untreated eMSC. (a) PPI network for the induced genes. The network contains clusters related to cell cycle, proliferation, transcription, translation, and chromatin remodeling. It shows that SHS-survived eMSC have more active transcriptome than untreated cells. Large TP53-regulated gene cluster with many hub proteins related to tumor suppression (TP53, STK11, CDKN1a, CDKN2A, TSC2) and excision DNA repair (PARP1, SP1, PML and ERCC2) indicates that SHS-survived eMSC trigger stress protection. (b) PPI network for the inhibited genes-B containing gene cluster involved in DNA damage checkpoint coordinated by ATM, ATR, BRCA1, CHK1, and gene cluster related to chromosome segregation demonstrates DNA instability in SHS-survived eMSC. Two gene clusters related to HRAS and growth factor signaling regulated by HRAS and KRAS oncogenes and YES1, EGFR1, ERBB3, and mTOR growth factors point to the weakened prooncogenic signaling. Overall, these data are in good agreement with chromosome instability in SHS-survived cells. The network was constructed using STRING server at interaction confidence > 0.9. Clusterization was done with K-means clustering. Hub proteins, that is, the ones having more than 5 connections, are marked with multicolor large buttons; node proteins are indicated with plain small buttons.

To obtain detailed PPI data, we constructed separate networks for genes that were induced or inhibited in SHS-survived versus control eMSC by more than 8-folds. For this purpose, we took interactions with the highest confidence (*S* > 0.9, Figure 3(a)) and extracted the whole connected components. The network of induced genes contained larger clusters and more connections compared to the network of the inhibited genes (Figures 3(a) and 3(b)), confirming a modular character of transcriptome activation in SHS-survived cells compared to control. Also, in accordance with the analysis of GO categories and BioSystem pathways, the clustering analysis with cluster functional characterization by gene module enrichment analysis using STRING database revealed many clusters. The main clusters were related to cell cycle (G1-S transition, S-phase, and metaphase), growth, protein synthesis, transcription, DNA repair, apoptosis, and tumor suppression in the induced network. The clusters related to signaling by oncogenes of RAS, MAPK, EGFR, JUN families, DNA damage checkpoint (ATM, ATR, BRCA1, and CHEK1), as well as olfactory and adrenal receptors, and negative metabolism regulation were found in the inhibited network (Figure 3(b)). Notably, the decreased activity of DNA damage checkpoint proteins may trigger unscheduled initiation of DNA replication [53]. Overall, the data of network analysis are in good agreement with the data of gene module examination and indicate that SHS-survived cells differ from control cells by gene expression and metabolic activation.

### 3.3. Transcriptome-Wide Analysis of Transformation-Related Features

#### 3.3.1. CD Marker Expression Pattern

**Figure 4:**
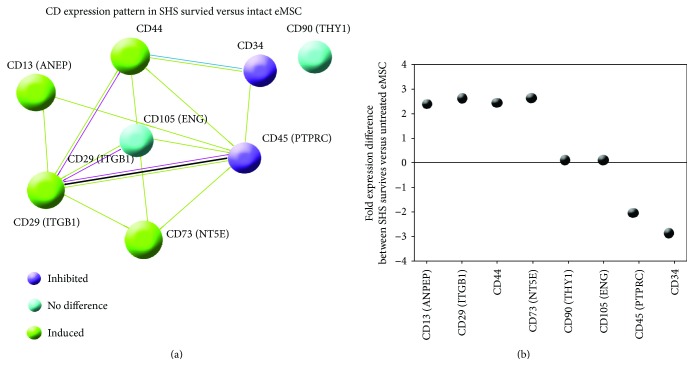
CD marker expression pattern in SHS-survived eMSC. (a) PPI network illustrating interactions between CD markers at interaction stringency *S* = 0.7 and the network constructed with STRING server. (b) Expression difference of CD markers between SHS-survived versus intact eMSC.

One of the most important questions of studies concerning stem cell research and implications in regenerative medicine is whether these cells can provide safety from oncogenic transformation. To investigate transformation-related features, we first examined whether HS-survived eMSC maintain appropriate CD marker expression pattern, that is, express mesenchymal stem cell-specific multipotent CD markers (CD13, CD29, CD44, CD73, CD90, and CD105) and do not express hematopoietic CD markers (CD34 and CD45) [18]. From the results of NGS data analysis, SHS-survived eMSC increased expression of CD13 (ANPEP) (2.3 folds), CD29 (ITGB1) (2.6 folds), CD44 (by 2.4 folds), and CD73 (NT5E) (2.8 folds) did not change the expression of CD90 (THY1) and CD105 (ENG) and decreased the expression of hematopoietic markers CD34 (SPN) (by 3.6 folds) and CD45 (PTPRC) (by 2.1 folds) compared to control (see Figures 4(a) and 4(b)) suggesting that these cells have normal phenotype inherent for nontransformed eMSC. This result is in good agreement with our previous data obtained by the method of immunophenotyping with eMSC after the same treatment [15].  Figure 4(a) illustrates protein interaction network for these CD markers indicating that all of them (with the exception of CD90 (THY1)) are interconnected by tight functional links. However, this encouraging result still does not guarantee safety. Recent studies indicated that human mesenchymal stem cells of bone marrow that were experimentally transformed with hTERT continued to express the normal CD pattern [18].

Therefore, to further examine manifestation of cancer-related features in SHS-survived eMSC compared to control, we checked our data according to the criteria of Hanahan-Weinberg describing the hallmarks of cancer [57]. These criteria include (1) increased and sustainable proliferation unbalanced by senescence and apoptosis, (2) growth suppressor inhibition, (3) cell death resistance, (4) replicative immortality, (5) angiogenesis, and (6) invasion and metastasis. It is worth noting that currently, Hanahan and Weinberg's work has more than 11,000 citations and thus can be considered as a classical blueprint for identification of transformation-related traits.

To characterize the data with regards to Hanahan-Weinberg criteria, we first revealed enriched GO categories and BioSystem pathways for 8724 genes (differentially expressed at 2.0-fold threshold) and searched among these modules the ones related to cell cycle, proliferation, growth, pluripotency, multipotency, cell senescence, apoptosis, oncogenes, tumor suppression, telomere extension and maintenance, angiogenesis, metastasis, and epithelial to mesenchymal transition (EMT). Significance levels were set at *p* < 0.01 and *q* < 0.15 (Supplementary Table 2). These thresholds were chosen on the ground of recommendations of GSEA group and other authors [58, 59]. Also, we performed a detailed functional analysis of genes unified by clusters in protein interaction networks (Figures 4(a) and 4(b)). Below, we provide a brief description of the obtained results.

### 3.4. Proliferation-Senescence Axis Characterizing the Hallmark “Ability to Maintain Sustainable Cell Proliferation Unbalanced by Senescence and Apoptosis”

One of the most fundamental hallmarks of transformed cells is their ability to sustain proliferation by uncontrollable release of growth and proliferation-promoting signals, which is followed by the disruption of signaling attenuating proliferation [57]. To evaluate the activity of proliferation-related gene modules, we selected GO categories and BioSystem pathways enriched for differentially expressed genes containing in their title terms related to proliferation and cell cycle (Figure 2, Supplementary Table 2). Compared to control, SHS-survived eMSC showed increased activity of modules coordinating the first half of cell cycle including G1-S transition, S-phase, DNA replication, chromosome segregation, and cell cycle regulation (Figure 2, Supplementary Table 1). Also, the examination revealed several fold increase of APC/CDC20 and PLK1 signaling. Deregulation of these pathways may trigger aneuploidy by means of premature chromosome separation [60, 61].

The PPI network consisting of induced genes (Figure 3(a)) contained a large cell cycle-related cluster with hubs and nodes related to S-phase (CCNB1 and CCNB2, CCND3, CDC16, MCM3, MCM7, and CDC25) and chromosome separation (PLK1, ANAPC5, 7, CDC20, and TUBA1A). Consistently, the network of downregulated genes contained a cluster of regulators of sister chromatid cohesion containing PDS5A, CENPE, BUB3, and AURKB (Figure 3(b)). Thus, the gene module and protein interaction network analysis points to inappropriate chromosome segregation and confirm the results of G-banding revealed aneuploidy in SHS-survived cells.

To find out whether the induction of cell cycle-related modules was compensated by processes implicated in senescence and cell death, we selected the modules containing terms “senescence,” “aging,” and “cell death.” The analysis identified several induced modules related to senescence and a substantial amount of modules implicated in TP53 signaling (Figure 2, Supplementary Table 1).

The PPI network of induced genes related to senescence contained a big cluster with TP53 protein as a hub and important regulators related to TP53 stabilization, and aging (CDKN2A (p16), CDKN2AIP (p16 interacting protein), and CDKN1A (p21)) as targets (Figure 3(a)). No modules and network clusters of senescence were found to be downregulated. Thus, our data indicate that manifestations of increased proliferative signaling and DNA instability are well balanced by prosenescence pathways in SHS-survived eMSC suggesting it does not meet the first Hanahan-Weinberg criterion entitled as “sustainable proliferation unbalanced by senescence and apoptosis” (Figure 3).

### 3.5. Proapoptotic and Prooncogenic Signaling Balance Characterizing the Hallmarks “Evading Growth Suppressors” and “Cell Death Resistance”

In contrast to normal cells, cancer cells evade programs of negative regulation of proliferation executed mostly by tumor-suppressing genes and pathways [57]. The most important of them are presented by TP53 gene and their signaling. Compared to control, SHS-treated eMSC show the induction of pathways coordinated by TP53 and other important pathways promoting tumor suppression (Figure 2, Supplementary Table 1). Also, the important finding was the strong activation of the BioSystem pathway “validated targets of C-MYC transcriptional repression.” The upregulation of this pathway including genes that are usually silent when MYC is active indicating that eMSC possess particular system protecting against MYC induction even under severe stress caused by DNA instability and confirms our NGS results pointing to the absence of MYC expression in SHS-untreated and SHS-treated cells. This result is surprising because DNA instability associated with aneuploidy, polyploidy, or chromosome breakages usually enhances cancer phenotype and activates C-MYC [26, 62–65].

**Figure 5:**
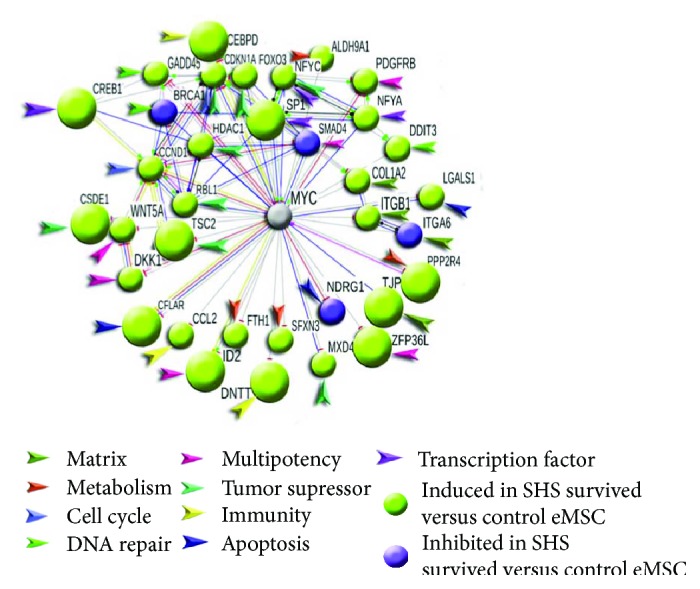
Gene composition of BioSystem pathway “validated targets of C-MYC transcriptional repression.” The figure illustrates gene functional distribution and expression gradient through the pathway and indicates that all genes (31 induced and 4 inhibited) are interconnected with MYC as genes that are directly repressed by MYC. The repressing type of interactions is indicated with small red solid lines (bricks) near buttons. MYC silence is indicated with a *grey button*. The network is constructed using STRING server at interaction stringency *S* = 0.9.

To examine gene functional distribution and expression gradient through the pathway “validated targets of C-MYC transcriptional repression,” we constructed protein interaction network containing all 35 proteins this pathway.  Figure 5 indicates that all genes (31 induced and 4 inhibited) are interconnected with MYC as genes that are directly repressed by MYC. Most of the induced genes are related to DNA repair, tumor suppression, and multipotency. Thus, protein interaction network indicates that “validated targets of C-MYC transcriptional repression” pathway contains mainly activated genes, which should be repressed if MYC was activated. This confirms that HS-survived cells may have specific barrier against MYC activation. Thus, our data indicate that HS-survived eMSC do not have death resistance.

It is well established that cell death resistance may be triggered by impaired proapoptotic and cell death-related signaling accompanied by elevated signaling of oncogenesis [57]. From our results, SHS-survived eMSC demonstrated the upregulation of modules involved in apoptosis and cell death (Figure 2, Supplementary Table 1).

To investigate signaling implicated in oncogenesis, we selected gene modules with terms related to cancer, transformation, tumors of various origins, and well-known oncogenes from different families of growth factors, serine/threonine, and tyrosine kinases (ABL, EGFR, ERBB, JAK, MAPK, and AKT), GTPases (RAS), and transcription factors (MYC, JUN, and FOS). The selection yielded no induced pathways. In contrast, the inhibited prooncogenic modules were quite numerous evidencing in favor of nontransformed state of SHS-survived eMSC (Figure 2, Supplementary Table 1). Thus, compared to untreated eMSC, SHS-survived eMSC demonstrated the downregulation of pathways implicated in signaling coordinated by RAS, ERBB, and MAPK.

Notably, our data revealed no expression of a master regulator of PI3K signaling PKB alpha (AKT1) and MYC oncogene in untreated eMSC and in SHS-survived ones. The important role of RAS, EGFR, ERBB, MAPK, AKT1, and MYC in transformation is currently well established and was reviewed in [57]. Also, the activity of tumor marker carcinoembryonic antigen-related cell adhesion molecule (CEACAM5) was decreased by more than 16-folds as well as the other proteins of CEACAM family. The implication of CEACAM family proteins in cancer progression and metastasis has been recently underlined by Bajenova and coauthors [66, 67]. The authors revealed the tight interaction between CEACAM proteins and MAPK, TGF-beta, and EMT pathways.

In accordance with the results of gene module analysis pointing to the nontransformed state of SHS-survived eMSC, the data of the examination of general PPI network constructed for the induced and inhibited genes revealed no oncogene containing clusters among the network of the induced genes and revealed three tight transformation-related clusters implicated in FOXO, HRAS, and EGFR signaling among the network of the inhibited genes (Figure 2, Supplementary Table 1).

### 3.6. Activity of Telomere- and Telomerase-Related Gene Modules as Indicators of the Hallmark “Replicative Immortality”

Gene modules implicated in replicative immortality were searched by the titles containing terms related to telomere and telomerase. The search gave no results. To additionally verify the absence of features related to “replicative immortality,” we analyzed genes participating in telomere extension, protection, and maintenance. The information about these genes were found in the TeloPIN database describing telomeric proteins and interaction network in mammalian cells [68]. The examination of genes hTERT, TRF1, TRF2, TIN2, RAP1, POT1, and TTP1 indicated that SHS-treated eMSC express only two genes (POT1 and TTP1) providing telomere protection. These genes were induced by 15.6- and 12.4-folds in the treated versus untreated eMSC. Other genes were not expressed. The absence of telomerase activity was found in other types of human mesenchymal stem cells including hematopoietic and nonhematopoietic stem cells such as neuronal, skin, adipose tissue, intestinal crypt, mammary epithelial, pancreas, adrenal cortex, and kidney [69].

### 3.7. Gene Modules Characterizing the Hallmarks “Angiogenesis” and “Invasion and Metastasis”

The examination of gene modules with terms related to angiogenesis in their titles gave no results. Cell program related to invasion and metastasis was inhibited. It is evident from the dramatically decreased activity of transcription factors of SNAI and ZEB families (Supplementary Table 1) that are known to regulate prometastatic program of EMT [70]. Also, there are no manifestations of N-cadherin to E-cadherin switching, which is also an important EMT feature [70]. Moreover, the pathways of “stabilization and expansion of the E-cadherin adherens junction” and “E-cadherin signaling in keratinocytes” were induced (Supplementary Table 1). Accordingly, the two processes related to migration, including “positive regulation of neuron migration” and “cell migration involved in gastrulation,” were downregulated confirming that the metastatic potential was not activated in SHS-survived eMSC (Figure 2, Supplementary Table 1).

### 3.8. Characteristic-Enabling Transformation

Besides the hallmarks of cancer [57], Hanahan and Weinberg identified characteristic-enabling transformation, including genome instability Warburg effect, inflammation, and avoided immune destruction. Of these characteristics, SHS-survived eMSC demonstrate only chromosome instability (Figure 1). In agreement with this observation, the transcriptome analysis identified features of chromosome missegregation and premature separation described above (Figures 2 and 3, Supplementary Table 1). Also, our data revealed features of DNA damage response modification, specifically, the increased activity of single-strand DNA repair and the impairment of double-strand DNA repair (DDR). The induction of modules related to DNA excision repair, TP53-dependent G1/S DNA damage checkpoint and network clusters containing TP53, TP53BP1, DDIT3, ERCC2, PARP1, and RFC1 DNA repair regulators was accompanied by the coordinated downregulation of the protein interaction network cluster containing ATM, CHEK1, BRCA1, and ATR (Figures 2 and 3, Supplementary Table 1). All these genes are well-known regulators of double-strand DNA damage sensing and repair and for the coordination of DNA replication checkpoint and appropriate S-phase entry (ATR and CHEK1) [71, 72]. Fortunately, the weakening of DDR is associated with compensatory induction of pathways involved in DNA-based excision repair, apoptosis, and senescence. The activation of these pathways (particularly the ones of single-strand DNA repair) may guard SHS-survived eMSC from transformation even despite prominent genomic instability [73]. Thus, our analysis revealed both flaws and strengths of eMSK SHS resistance and explained why these cells escape transformation despite extensive genomic instability.

The other cancer-enabling characteristic related to metabolic switches towards Warburg effect was not identified. Energy metabolism-related gene modules were activated and were implicated mainly to aerobic respiration and mitochondrial electron transport chain (Figure 2, Supplementary Table 1). Our data did not identify features of Warburg-like metabolic modifications, including the induction of modules of glycolysis and glutaminolysis and the inhibition of modules related to aerobic respiration. This result indicates that SHS-survived eMSC do not show transformation-related metabolic modification.

Gene modules implicated in transformation enabling characteristics related to inflammation and immune destruction were coordinately downregulated and were presented by modules of immunity mediated by B- and T-lymphocytes, immunoglobulines, interleukins, and adrenergetic receptor signaling (Figure 2, Supplementary Table 1), confirming the absence of these transformation-related characteristics in SHS-survived eMSC and their anti-inflammatory eMSC properties.

### 3.9. Outcome of Long-Term Cultured eMSC-Survived SHS

**Figure 6:**
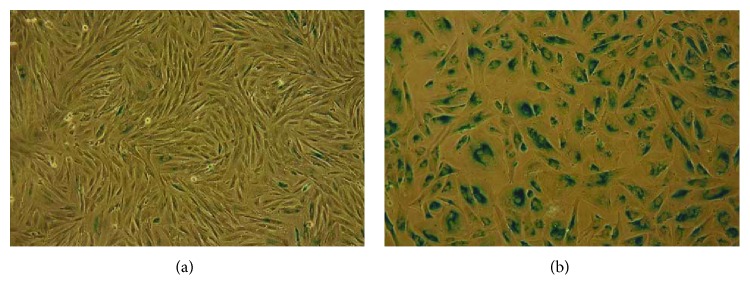
SA-beta-Gal staining of SHS-survived cells at the 6th (a) and 19th (b) passages.

SHS-survived eMSC analyzed at the 6th passage after HS were then expanded further. They were subcultured for another 19 passages and slowly died. During the passaging, these cells demonstrated gradually reduced proliferation and morphological abnormalities manifested in cell enlargement. In other words, these cells slowly entered into the replicative senescence state. These observations were supported by the results of SA-beta-Gal activity in cells at various passages. The enzyme activity is a marker of cellular senescence.  Figure 6 shows SHS-survived cells at the 6th (A) and 19th (B) passages after SHS exposure. It is seen that at the moment of karyotyping, next-generation sequencing and transcriptome functional analysis (passage 6) cells had typical fibroblast-like morphology and did not exhibit SA-beta-Gal activity (Figure 6(a)). At the 19th passage, Figure 6(b) exhibits cell enlargement and bright staining of SA-beta-Gal-positive cells that are hallmarks of the replicative senescence. These cells ceased proliferation and then died. So, in spite of genetic instability, SHS-survived cells remain mortal and nontransformed.

### 3.10. Comparison of Our Results with the Data on Transformed Bone Marrow Mesenchymal Stem Cells (bmMSC)

The detailed investigation of Hanahan-Weinberg hallmarks of cancer indicates that although SHS-survived eMSC show genomic instability, these cells can be considered as nontransformed cells. It is reasonable to compare our data with the data obtained with experimentally transformed mesenchymal stem cells. For this propose, we used the data on gene expression changes in human bone marrow mesenchymal stem cells after hTERT transformation [18]. We made gene-by-gene comparison of gene sets containing the most severely deregulated genes during hTERT-induced transformation of bone marrow MSC with our results. Of 123 genes, only 21 genes changed expression in the same direction in hTERT-transformed and SHS-survived eMSC cells, whereas 42 genes changed expression in opposite direction. The remaining 60 genes were expressed in control and hTERT-transformed cells and were not expressed in intact and SHS-survived eMSC (Supplementary Table 2).

**Figure 7:**
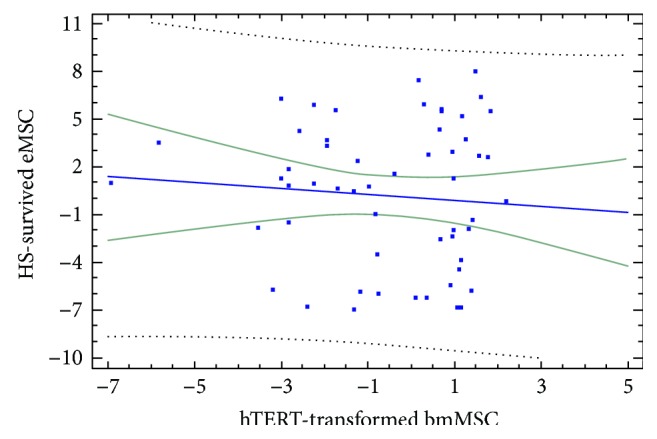
The absence of correlation between gene expression changes in hTERT-transformed bmMSC and SHS-survived eMSC (*r* = −0.10, *p* > 0.5).

The regression analysis revealed the absence of correlation between gene expression changes in hTERT-transformed bmMSC and SHS-survived eMSC (*r* = −0.10, *p* > 0.5, Figure 7). In accordance, the binomial test revealed the significant prevalence of the fraction of gene changing expression in bmMSC and eMSC in the opposite directions over the fraction of gene changing expression in the same direction (*p* < 10^−5^).

To further verify the absence of transformation features in SHS-survived eMSC, we compared the activity of gene modules enriching for above-said sets containing 22, 45, and 44 genes (Supplementary Table 3). The examination of the 45-gene set indicated that in contrast to hTERT-transformed bmMSC, SHS-treated eMSC show increased ability to differentiation and cell adhesion and decreased PI3K-AKT signaling. The analysis of the 22-gene set shows that both hTERT-transformed and SHS-survived eMSC increase proliferative potential after treatment. The investigation of the 44-gene set revealed the silence of cancer-related modules in SHS-survived cells compared to hTERT-transformed cells (Supplementary Table 3). Thus, the obtained results indicate that SHS-survived eMSC do not show features of transformation revealed in bmMSC after hTERT treatment.

## 4. Conclusion

Generally, our data indicated that compared to untreated cells, SHS-survived eMSC show higher genome instability, which exert global effect on gene expression and activity of gene modules related to essential biological pathways. The induction of energy metabolism, protein turnover, and transcription give SHS-survived cells beneficial properties helping them to overcome high-energy needs that are necessary to counteract aneuploidy and chromosome breakages. The evaluation of Hanahan-Weinberg criteria of transformation entitled as “hallmarks of cancer” and “cancer-enabling characteristics” did not reveal features of malignancy even despite genomic instability in eMSC. This result indicates that SHS-survived eMSC possess strong defense system protecting them against transformation. Indeed, we identified several lines of defense. The first line is provided by the ability of eMSC to decrease prooncogenic pathway activity (specifically, pathways regulated by oncogenes of RAS, Pi3k, MAPK, and ERBB families) in response to DNA damage and aneuploidy. The second line originates from the coordinated induction of tumor-suppressing pathways, including the pathways of TP53, p21 (CDKN1A), and p16 (CDKN2A) signaling, and pathways implicated in DNA repair. The third line is the increased protection against of single-strand breakages of DNA seen from the activation of pathways related to DNA excision and mismatch repair. Also, we found the silence of oncogenes MYC, AKT1/PKB, and hTERT telomerase that may further increase protection against transformation. Overall, our data suggest that despite genetic instability, SHS-survived eMSC do not undergo transformation and entered replicative senescence after prolonged expansion in culture, confirming their mortality and oncological safety.

## Supplementary Materials

Supplementary materials Supplementary Table 1. Gene modules enriching for genes that are significantly induced or repressed in SHS-survived vs control eMSC. Supplementary Table 2. Comparion of gene expression changes in SHS survuved vs untreated eMSC (present study) and in hTERT transformed vs intact bone marrow MSC (^∗^the data from Takeuchi et al, 2015). the data from Takeuchi et al, 2015). Supplementary Table 3. Comparison of gene module activity enriching for 22, 45 and 44 gene sets in hTERT and SHS treated bmMSC and eMSC.
