# Supplementary material for: Molecular Genetic Analysis of Human Endometrial Mesenchymal Stem Cells That Survived Sublethal Heat Shock
Source: Stem Cells Int. 2017 Dec 10;2017:2362630. doi: 10.1155/2017/2362630 (PMC5742502; doi:10.1155/2017/2362630)
Supplement: Supplementary materials — Supplementary Table 1. Gene modules enriching for genes that are significantly induced or repressed in SHS-survived vs control eMSC. Supplementary Table 2. Comparion of gene expression changes in SHS survuved vs untreated eMSC (present study) and in hTERT transformed vs intact bone marrow MSC (∗the data from Takeuchi et al, 2015). the data from Takeuchi et al, 2015). Supplementary Table 3. Comparison of gene module activity enriching for 22, 45 and 44 gene sets in hTERT and SHS treated bmMSC and eMSC. [file 2362630.f1.pdf]

Supplementary Table 1: Gene modules enriching for genes that are significantly induced or repressed in SHS-survived vs control eMSC

Pathway or GO ID	Pathway or GO category name	F_D*	№**	P-value	q-value
Cell Cycle					
REACT_6954	Activation of APC/C and APC/C:Cdc20 mediated degradation of mitotic proteins	2.1	55	2.00E-04	0.01671
KEGG__hsa03030	DNA replication	2.992	21	1.60E-03	0.06307
REACT_2014	Synthesis of DNA	1.651	70	4.00E-04	0.00879
REACT_150471	Separation of Sister Chromatids	1.623	103	2.00E-04	0.01671
REACT_6781	APC/C:Cdc20 mediated degradation of mitotic proteins	2.058	54	5.00E-04	0.03165
REACT_6761	APC/C:Cdh1 mediated degradation of Cdc20 and other APC/C:Cdh1 targeted proteins in late mitosis/early G1	1.957	54	7.00E-04	0.0375
138077__PID	Polo-like kinase signaling events in the cell cycle	3.279	3	1.40E-03	0.03944
REACT_899	S Phase	1.425	82	4.00E-03	0.09831
REACT_6785	Autodegradation of Cdh1 by Cdh1:APC/C	1.852	48	4.50E-03	0.10331
138051__PID	EPO signaling pathway	3.198	14	6.90E-03	0.12427
KEGG__M00693	Cell cycle - G2/M transition	4.152	5	7.40E-03	0.12883
REACT_6820	APC/C:Cdc20 mediated degradation of Cyclin B	3.125	14	8.20E-03	0.13396
REACT_829	Regulation of DNA replication	1.492	58	9.50E-03	0.14487
REACT_22172	Chromosome Maintenance	2.085	30	1.00E-02	0.14922
GO:0090068	positive regulation of cell cycle process	1.003	152	5.30E-03	0.04852
GO:0009262	deoxyribonucleotide metabolic process	2.83	19	4.40E-03	0.15715
GO:1901988	negative regulation of cell cycle phase transition	1.234	105	3.30E-03	0.12959
Senescence and apoptosis					
REACT_169274	Cellular Senescence	1.205	81	1.45E-03	0.04092
REACT_268456	Programmed Cell Death	1.075	118	8.30E-03	0.13396
REACT_13648	Regulation of Apoptosis	1.679	42	1.37E-02	0.06883
REACT_578	Apoptosis	1.075	118	8.30E-03	0.13396
Tumor supression					
137939__PID	Direct p53 effectors	1.925	80	6.90E-03	0.05043
REACT_355377	Transcriptional Regulation by TP53; TP53 Regulates Metabolic Genes	1.901	52	1.70E-03	0.07427
REACT_1625	p53-Dependent G1/S DNA damage checkpoint; p53-Dependent G1 DNA Damage Response	1.544	50	7.10E-03	0.08427
REACT_309	Stabilization of p53	1.539	51	1.97E-02	0.08505
GO:0072331	signal transduction by p53 class mediator	1.378	88	1.50E-04	0.047
GO:0072332	intrinsic apoptotic signaling pathway by p53 class mediator	2.206	29	6.00E-03	0.09048
137957__PID	LKB1 (STK11) signaling events	2.814	26	1.40E-03	0.0585
169353_PID	Validated targets of C-MYC transcriptional repression	1.911	35	1.20E-03	0.07132

WP28	Selenium Metabolism and Selenoproteins	2.679	24	3.30E-03	0.08839
REACT_12436	ERKs are inactivated	2.714	10	4.98E-03	0.09532
REACT_268323	Hedgehog 'off' state	1.528	62	7.10E-03	0.1257
Oncogenic pathways					
REACT_118778	Activation of RAS	-3.07	4	4.21E-03	0.05877
138016__PID	ErbB receptor signaling network	-3.47	9	8.22E-03	0.07742
545371__BIOCYC	MAP kinase cascade	-2.28	2	2.40E-03	0.08396
REACT_20546	Ras activation uopn Ca2+ infux through NMDA receptor	-2.04	13	5.27E-03	0.08703
REACT_121096	EGFR Transactivation by Gastrin	-3.16	8	5.72E-03	0.0613
E-cadherin signaling					
138005__PID	E-cadherin signaling in the nascent adherens junction	1.814	23	5.80E-03	0.08116
138002__PID	E-cadherin signaling in keratinocytes	2.395	11	7.30E-03	0.08155
Cell migration					
GO:0042074	cell migration involved in gastrulation	-3.72	8	1.73E-03	0.08571
GO:2001224	positive regulation of neuron migration	-3.79	7	1.36E-03	0.0927
Metabolism					
REACT_6305	Respiratory electron transport, ATP synthesis by chemiosmotic coupling, and heat production by uncoupling proteins.	2.335	81	0	0
REACT_71	Gene Expression	1.749	733	0	0
REACT_17015	Metabolism of proteins	1.194	486	0	0
REACT_111083	The citric acid (TCA) cycle and respiratory electron transport	2.047	109	0	0
REACT_22393	Respiratory electron transport	2.083	65	1.00E-04	0.01229
WP623	Oxidative phosphorylation	2.89	43	1.00E-04	0.01229
REACT_1014	Translation	1.457	137	2.00E-04	0.01671
REACT_125	Processing of Pre-mRNA	1.737	104	2.00E-04	0.01671
WP1785	DNA damage response and repair proteins	1.693	51	1.00E-02	0.04222
WP1530	miRNA Regulation of DNA Damage Response	2.631	5	1.56E-03	0.0757
REACT_1893	Base Excision Repair; Resolution of Abasic Sites (AP sites)	2.766	16	1.07E-03	0.08002
KEGG__hsa03410	Base excision repair	2.406	17	2.55E-03	0.08252
REACT_1826	Nucleotide Excision Repair	1.782	31	4.40E-03	0.08441
137918__PID	Stabilization and expansion of the E-cadherin adherens junction	1.528	28	6.95E-03	0.08488
REACT_216	DNA Repair	1.143	83	2.01E-02	0.08531
REACT_383	DNA Replication	1.503	74	3.00E-03	0.08585
REACT_169436	Oxidative Stress Induced Senescence	1.356	51	5.92E-02	0.09123
REACT_111119	Antigen processing-Cross presentation	1.632	60	4.50E-03	0.10331
REACT_163646	SUMO is transferred from E1 to E2 (UBE2I, UBC9)	5.015	6	4.50E-03	0.10331
REACT_163823	SUMOylation	1.813	49	4.10E-03	0.10383
REACT_6759	Formation of ATP by chemiosmotic coupling	3.279	14	5.00E-03	0.10872
REACT_6261	Abortive elongation of HIV-1 transcript in the absence of Tat	2.8	19	4.90E-03	0.10872
REACT_264135	Organelle biogenesis and maintenance	0.894	182	5.10E-03	0.10872
KEGG__hsa04962	Vasopressin-regulated water reabsorption	2.339	29	4.90E-03	0.10872

WP229	Irinotecan Pathway	-4.91	6	5.10E-03	0.10872
KEGG__hsa00230	Purine metabolism	1.234	99	4.80E-03	0.10872
KEGG__M00158	F-type ATPase, eukaryotes	3.279	14	5.00E-03	0.10872
KEGG__hsa03010	Ribosome	1.208	114	4.20E-03	0.11796
REACT_11123	Membrane Trafficking	1.169	121	4.30E-03	0.11839
KEGG__hsa04380	Osteoclast differentiation	1.453	66	6.00E-03	0.12053
KEGG__M00340	Proteasome, 20S core particle	3.134	15	6.00E-03	0.12053
WP1001	Peptide GPCRs	-7.18	2	5.90E-03	0.12053
REACT_2243	Assembly of the pre-replicative complex	1.638	55	6.20E-03	0.12105
REACT_268156	Degradation of GLI1 by the proteasome	1.79	47	6.20E-03	0.12105
REACT_11063	Degradation of beta-catenin by the destruction complex	1.543	60	6.90E-03	0.12427
REACT_15452	Peptide hormone biosynthesis	-7.13	2	6.90E-03	0.12427
REACT_15398	Glycoprotein hormones	-7.13	2	6.90E-03	0.12427
169346__PID	Regulation of RAC1 activity	2.594	21	6.70E-03	0.12427
KEGG__hsa03060	Protein export	2.672	19	6.70E-03	0.12427
REACT_355474	RHO GTPase Effectors	0.99	148	7.10E-03	0.1257
WP51	Regulation of Actin Cytoskeleton	1.235	91	7.30E-03	0.12816
REACT_2099	Eukaryotic Translation Initiation; Cap-dependent Translation Initiation	1.135	107	7.50E-03	0.12949
KEGG__hsa03008	Ribosome biogenesis in eukaryotes	1.807	49	5.00E-03	0.12983
REACT_355604	Vesicle-mediated transport	1.009	140	7.70E-03	0.13036
WP455	GPCRs, Class A Rhodopsin-like	-1.52	58	7.80E-03	0.13036
REACT_160130	Diseases associated with visual transduction; Retinoid cycle disease events	-7.09	2	7.80E-03	0.13036
REACT_163725	SUMO is proteolytically processed	5.13	5	7.70E-03	0.13036
REACT_268133	Mitochondrial translation elongation	1.578	54	8.10E-03	0.13396
WP1539	Angiogenesis	3.227	13	8.40E-03	0.13396
REACT_169208	Glycogen synthesis	3.429	11	8.40E-03	0.13396
138004__PID	Alternative NF-kappaB pathway	4.548	4	8.50E-03	0.13453
WP2035	FSH signaling pathway	2.769	17	8.90E-03	0.1398
PWY-7224	purine deoxyribonucleosides salvage	4.27	7	9.10E-03	0.14082
HUMAN_PWY-53	sulfate activation for sulfonation	3.904	2	9.60E-03	0.14533
REACT_931	Ribosomal scanning and start codon recognition	1.575	52	1.06E-02	0.15002
REACT_16973	Orexin and neuropeptides FF and QRFP bind to their respective receptors	-4.14	3	1.03E-02	0.15002

Transcription	GO module name				
GO:0000398	mRNA splicing, via spliceosome	1.752	152	0	0
GO:0044237	cellular metabolic process	0.301	4234	0	0
GO:0048193	Golgi vesicle transport	1.252	155	0	0
GO:0008104	protein localization	0.528	993	0	0
GO:0033036	macromolecule localization	0.559	1150	0	0
GO:0010604	macromolecule metabolism	0.433	1371	0	0
GO:0009059	macromolecule biosynthesys	0.387	1849	0	0
GO:0044267	cellular protein metabolic process	0.477	1876	0	0
GO:0043484	regulation of RNA splicing	1.983	59	9.00E-05	0.00024
GO:0008380	RNA splicing	1.138	226	1.00E-04	0.01151
GO:0006397	mRNA processing	1.131	255	1.00E-04	0.01151
GO:0090304	nucleic acid metabolic process	0.334	1959	1.00E-04	0.01151
GO:0006974	cellular response to DNA damage stimulus	1.834	401	2.00E-04	0.0125

GO:0002449	lymphocyte mediated immunity	-2.1	51	5.14E-04	0.0125
GO:0055114	oxidation-reduction process	0.658	508	5.00E-04	0.03666
GO:0009893	positive regulation of metabolic process	0.3	1748	1.20E-03	0.06542
GO:0002443	leukocyte mediated immunity	-1.39	65	1.33E-03	0.08849
GO:0019724	B cell mediated immunity	-2.28	28	9.00E-04	0.09699
GO:0051340	regulation of ligase activity	1.405	86	2.60E-03	0.1001
GO:1903321	negative regulation of protein modification by small protein conjugation or removal	1.378	90	2.60E-03	0.11048
GO:0019941	modification-dependent protein catabolic process	0.81	275	2.60E-03	0.11048
GO:0051603	proteolysis involved in cellular protein catabolic	0.76	302	2.60E-03	0.11048
GO:0065009	regulation of molecular function	0.326	1319	2.60E-03	0.11048
GO:1903362	regulation of cellular protein catabolic process	0.987	185	2.70E-03	0.11186
GO:0043632	process	0.772	281	2.70E-03	0.11186
GO:0036211	protein modification process	0.335	1351	2.70E-03	0.11186
GO:0006464	cellular protein modification process	0.335	1351	2.70E-03	0.11186
GO:0043603	cellular amide metabolic process	0.656	391	2.90E-03	0.11837
GO:1901566	organonitrogen compound biosynthetic process	0.524	590	2.90E-03	0.11837
GO:0034504	protein localization to nucleus	1.322	90	3.10E-03	0.12469
GO:0006612	protein targeting to membrane	1.067	145	3.10E-03	0.12469
GO:0045934	negative regulation of nucleobase-containing compound metabolic process	0.488	646	3.10E-03	0.12469
GO:0019229	regulation of vasoconstriction	-2.57	26	3.20E-03	0.12748
GO:0071806	protein transmembrane transport	2.425	30	3.20E-03	0.12748
GO:0032224	positive regulation of synaptic transmission,	-7.64	2	3.30E-03	0.12959
GO:0009394	2'-deoxyribonucleotide metabolic process	3.356	15	3.30E-03	0.12959
GO:0044802	single-organism membrane organization	0.585	435	3.40E-03	0.13227
GO:0009892	negative regulation of metabolic process	0.347	1234	3.40E-03	0.13227
GO:0046850	regulation of bone remodeling	-3.69	12	3.50E-03	0.13365
GO:0006706	steroid catabolic process	-3.68	13	3.50E-03	0.13365
GO:0030163	protein catabolic process	0.691	333	3.60E-03	0.13621
GO:0006451	translational readthrough	6.366	3	3.80E-03	0.1412
GO:0001514	selenocysteine incorporation	6.366	3	3.80E-03	0.1412
GO:1903322	positive regulation of protein modification by small protein conjugation or removal	1.184	112	3.90E-03	0.14427
GO:0070194	synaptonemal complex disassembly	6.918	2	4.10E-03	0.15032
GO:0045110	intermediate filament bundle assembly	-6.05	4	4.30E-03	0.15424
GO:0010875	positive regulation of cholesterol efflux	-4.58	7	4.30E-03	0.15424

F_D* Log2 of average fold difference for genes involved in a pathway in SHS survived vs untreated eMSC
№** Gene number in a pathway

Supplementary Table 2. Comparion of gene expression changes in SHS survuved vs untreated eMSC (present study) and in hTERT transformed vs intact bone marrow MSC (*the data from Takeuchi et al, 2015)

Genes that changed expression in the same direction in hTERT-transformed and SHS-survived eMSC cells (the 22 gene list)

Gene symbc	Gene annotation	**F_ D in eMSC	***F_D in bmMSC
ATAD2	ATPase family, AAA domain containing 2	2.693744962	4.82
PIK3CA	phosphatidylinositol-4,5-bisphosphate 3-kinase catalytic subunit alpha	5.637177442	1.95
DAB2IP	DAB2 interacting protein	-5.911036602	-3.28
PRKDC	protein kinase, DNA-activated, catalytic polypeptide	0.815980235	4.3
XRCC5	X-ray repair complementing defective repair in Chinese hamster cells 5	1.66511122	3.4
FRY	FRY microtubule binding protein	-6.992232987	3.87
MARCKSL1	MARCKS-like 1	-3.5547421	-2.1
PTTG1	pituitary tumor-transforming 1	3.701774085	3.59
RAD21	RAD21 cohesin complex component	4.271614407	1.97
CCNB1	cyclin B1	5.898624955	1.31
CDK4	cyclin-dependent kinase 4	2.709958339	1.5
CIZ1	1	7.944973164	4.51
E2F1	E2F transcription factor 1	5.637177442	2
XAF1	XIAP associated factor 1	-1.80147703	-10.5
IGF1R	insulin like growth factor 1 receptor	5.150563358	3.26
IGF2BP2	insulin like growth factor 2 mRNA binding protein 2	6.381244889	2.1
PDGFB	platelet-derived growth factor beta polypeptide	5.447669658	6.2
MSX2	msh homeobox 2	-6.766634504	10.1
FLT1	fms-related tyrosine kinase 1	2.887719472	2.6
PDGFB	platelet-derived growth factor beta polypeptide	5.447669658	6.2
MTA1	metastasis associated 1	-4.114305969	-3.21
SOX4	SRY-box 4	-5.97747568	-2.24

Genes that changed expression in opposite direction in hTERT-transformed and SHS-survived eMSC cells (the 45 gene list)

Gene symbc	Gene annotation	**F_ D in eMSC	***F_D in bmMSC
RAI2	transferrin receptor (CD71)	-0.84562321	42.5
MYBL2	v-myb avian myeloblastosis viral oncogene homolog-like 2	-1.303399719	4

RRAS2	related RAS viral (r-ras) oncogene homolog 2	5.447669658	-3.2
HIC1	hypermethylated in cancer 1	1.369160022	-3.2
RASA4	RAS p21 protein activator 4	4.243219397	-8
BRCA1	breast cancer 1	-1.914790568	3.8
RAD9A	RAD9 checkpoint clamp component A	-3.882713009	3.1
TREX1	three prime repair exonuclease 1	0.673443916	-3.5
CENPE	centromere protein B	-0.996819457	2.7
CCND2	cyclin D2	-6.833889492	2.9
BCL2L11	BCL2-like 11 (apoptosis facilitator)	-6.248087035	2
BCL2L12	BCL2-like 12 (proline rich)	-6.294098098	2.3
CASP10	caspase 10	5.532679563	-7.4
APC	adenomatous polyposis coli	-5.540096982	2.5
CTNNB1	catenin beta 1	1.526349958	-1.6
DKK3	dickkopf WNT signaling pathway inhibitor 3	-0.770549326	2.25
WNT5A	wingless-type MMTV integration site family member 5A	2.056581454	-8.47
CSF2	colony stimulating factor 2 (granulocyte-macrophage)	-6.854057627	2.3
EIF4B	eukaryotic translation initiation factor 4B	-0.219956085	5.9
F2RL1	coagulation factor II (thrombin) receptor	-0.166800347	4.8
TFRC	transferrin receptor	-5.846085596	3.8
ACTA2	actin, alpha 2, smooth muscle, aorta	1.904361494	-8.4
HMGA2	high mobility group AT-hook 2	-4.429339353	3.06
POSTN	periostin, osteoblast specific factor	3.565235757	-14.3
TAGLN	transgelin	1.811100282	-7.5
THY1	Thy-1 cell surface antigen	0.009548314	-5.9
CDH2	cadherin 2, type 1, N-cadherin (neuronal)	-0.524072578	1.96
COL4A5	collagen, type IV, alpha 5	-5.569748468	38.2
FN1	fibronectin 1	0.921282332	-8.4
ICAM1	intercellular adhesion molecule 1	0.837115163	-6.4
ITGA6	integrin subunit alpha 6	-1.062067995	2.65
MCAM	melanoma cell adhesion molecule	-5.729456131	-10.6
VCAN	versican	3.644240224	-7.3
FBN2	fibrillin 2	-0.101800749	4.19
FGF2	fibroblast growth factor 2 (basic)	5.839729038	-3.1
NRP1	neuropilin 1	-1.506849222	-1.52
THBS1	thrombospondin 1	1.284946107	-8.24
VEGFC	vascular endothelial growth factor C	3.339996507	-4.2
MMP14	matrix metallopeptidase 14	1.825987452	-4.6
MMP3	matrix metallopeptidase 3	1.623256589	35
TIMP1	TIMP metallopeptidase inhibitor 1	1.225146379	-4.2
CDH1	E-cadherin	2.456987546	-1.48
CDKN2B	cyclin D-dependent kinase (CDK4 or CDK6) inhibitor, p14 or p15	4.285698345	-25.2

ELN	elastin	2.136954245	-14.8
MTSS1	metastasis suppressor 1	1.338877562	-5.1

Genes that are espressed in hTERT transformed and untreated bm MSC
and are silent genes in SHS survived and control eMSC (the 44 gene list)

Gene symbc	Gene annotation	**F_ D in eMSC	***F_D in bmMSC
BMI1	polycomb group ring finger protein	No_Expression	4.3
RHEB	Ras homolog, small GTPase protein	No_Expression	1.16
RHEBL1	Ras homolog	No_Expression	1.26
DBC1	inhibit SIRT1 function	No_Expression	-7.6
PYHIN1	pyrin and HIN domain family	No_Expression	2.7
RAP1GAP	GTPase activator for Ras-related protein (RAP1A)	No_Expression	-9.4
RB1CC1	enhancse RB1 gene expression	No_Expression	2.39
SFN	stratifin, stabilize p53	No_Expression	-2.8
TP63	p53 family	No_Expression	-3.1
MDC	mediator of DNA-damage checkpoint	No_Expression	3.95
XRCC3	maintain chromosome stability and repair DNA damage in HR	No_Expression	-2.6
CENPB	centromere-associated protei	No_Expression	-2.04
CETN3	centrin	No_Expression	3.03
ESPL1	separase, sister chromatid separation	No_Expression	2.75
MAD2L1	mitotic spindle assembly check point	No_Expression	6.45
RFC4	elongation of primed DNA templates by DNA polymerase delta	No_Expression	3.17
STAG2	a component of cohesin	No_Expression	3.68
CCNE2	G1/S specific cyclin E	No_Expression	2.59
CDK2	cyclin-dependent protein kinase for late G1 phase	No_Expression	1.78
CDKN1C	G1 cyclin-dependent kinase inhibitor, p57	No_Expression	-1.4
CDKN2AIPN	CDKN2A(p16)-interacting protein	No_Expression	2.5
BCL2	suppress apoptosis	No_Expression	2.05
HRK	harakiri, BCL2 interacting protein	No_Expression	-10.2
TNFRSF11B	Fas-like protein	No_Expression	-2.14
TNFRSF6B	tumor necrosis factor receptor, suppress cell death	No_Expression	4.5
DHH	desert hedgehog, activates stromal hedgehog effectors	No_Expression	3.44
FZD1	Wnt protein receptor; frizzled 1	No_Expression	-3.2
GLI1	transcriptional activator	No_Expression	1.41
HHIP	hedgehog interacting protein, negative regulators	No_Expression	-2.08
PTCH1	receptor for hedghog protein (Dhh, Shh, Ihh)	No_Expression	1.98

FGF14	fibroblast growth factor 14	No_Expression	5.1
NCBP2	nuclear cap binding protein	No_Expression	5.41
RAI2	transferrin receptor (CD71)	No_Expression	42.1
GPC5	glypican 5	No_Expression	135
LMOD1	smooth muscle leiomodin 1	No_Expression	-10.4
COL15A1	collagen, type XV, alpha 1	No_Expression	-0.76
ANGPT1	angiopoietin 1, important roles in vascular development	No_Expression	-4.1
F2RL1	fibrillin	No_Expression	4.76
KDR	type III receptor tyrosine kinase, receptors of the VEGF	No_Expression	2.95
CTSF	cathepsin F, lysosomal protease, implication in tulour invasion and metastasis	No_Expression	1.894
CTSH	cathepsin H, degradation of lysosomal protein	No_Expression	3.918
mmp1	matrix metallopeptidase 1	No_Expression	-8.29
mmp2	matrix metallopeptidase 2	No_Expression	-2.63
TIMP4	TIMP metallopeptidase inhibitor 4	No_Expression	-3.21

* The data for mostly deregulated genes in hTERT transformed vs intact bm MSC were taken from Takeuchi, et al, 2015 for comparison

Reference: Takeuchi M, Higashino A, Takeuchi K, Hori Y, Koshiba-Takeuchi K, Makino H, Monobe Y, Kishida M, Adachi J, Takeuchi J, Tomonaga T, Umezawa A, Kameoka Y, Akagi K. Transcriptional Dynamics of Immortalized Human Mesenchymal Stem Cells during Transformation. PLoS One. 2015. 2015 15;10(5):e0126562. doi: 10.1371/journal.pone.0126562. eCollection

** Expression fold difference (Log_2) in SHS survived eMSC vs control eMSC

*** Expression fold difference in hTERT transformed compared to intact bmMSC at the forth (last) styage of transformation

Supplementary Table 3.
Comparison of gene module activity enriching for 22, 45 and 44 gene sets in hTERT and SHS treated bmMSC and eMSC.

Pathway ID	Pathway description	Count in gene set	false discovery rate	Fold_1 hTERT transformed vs intact bm MSC	Fold_2 SHS survived vs intact eMSC
**Gene module enriching for genes that are expressed in hTERT transformed and control bmMSC and that are silent in control and experimental eMSC (60 genes)**					
GO:0002009	Morphogenesis of an epithelium	11	7.20E-05	1.87±0.7	0
KEGG:04110	Cell cycle	10	1.47E-08	1.41±0.6	0
KEGG:05200	Pathways in cancer	12	2.20E-08	1.64±0.5	0
KEGG:05205	Proteoglycans in cancer	10	9.25E-07	1.52±0.4	0
**Gene module enriching for genes that changed expression in the same direction in experiment vs control cells in hTERT transformed bmMSC and in SHS survived eMSC (21 genes)**					
GO:0010564	Regulation of cell cycle process	11	1.7E-05	0.34±0.2	0.28±0.2
GO:1901701	Cellular response to oxygen-	10	3.20E-05	0.39±0.1	0.31±0.2
**Gene module enrichment for genes that changed expression in opposite directions in experiment vs control cells in hTERT transformed bmMSC and in SHS survived eMSC (42 genes)**					
GO:0030154	Cell differentiation	16	3.19E-04	-1.78±0.3	0.46±0.2
GO:0030198	Extracellular matrix organization	11	4.99E-08	-1.4±0.5	0.7±0.2
GO:0040011	Locomotion	15	3.50E-07	-1.6±0.3	1.5±0.3
GO:0030155	Regulation of cell adhesion	10	1.20E-05	-2.3±1.8	0.68±0.2
GO:0045597	Positive regulation of cell	10	1.20E-04	-1.27±1.8	0.53±1.8
KEGG:04151	PI3K-Akt signaling pathway	13	5.40E-12	1.83±0.2	-1.21±0.3
KEGG:04510	Focal adhesion	7	6.69E-05	1.82±0.2	-1.34±0.3
